# Proactive prosociality in a cooperatively breeding corvid, the azure-winged magpie (*Cyanopica cyana*)

**DOI:** 10.1098/rsbl.2016.0649

**Published:** 2016-10

**Authors:** Lisa Horn, Clara Scheer, Thomas Bugnyar, Jorg J. M. Massen

**Affiliations:** Department of Cognitive Biology, University of Vienna, Vienna, Austria

**Keywords:** prosociality, group service paradigm, cooperative breeding

## Abstract

One of the contemporary hypotheses concerning the evolution of human altruism is the cooperative breeding hypothesis (CBH) which has recently been tested in non-human primates. Using a similar paradigm, we investigated prosociality in a cooperatively breeding corvid, the azure-winged magpie. We found that the magpies delivered food to their group members at high rates, and unlike other corvids, they did so without any cues provided by others. In two control conditions, the magpies stopped participating over time, indicating that they learned to discriminate prosocial tests from controls. Azure-winged magpies are thus the first birds that experimentally show proactive prosociality. Our findings are in line with the CBH; however, additional corvid species need to be tested in this promising paradigm.

## 1. Introduction

The evolution of altruism remains a highly debated topic, particularly because the first studies on prosociality in chimpanzees rendered negative results (e.g. [1]), and prosociality was subsequently considered a human hallmark. Prosociality, here defined as helping another individual at low or no cost to the self [1], has since been tested in a multitude of primates with sometimes positive, sometimes negative and sometimes mixed results per species (for a review, see [2]). One of the hypotheses that tries to explain the evolutionary reasons for this mixed pattern is the cooperative breeding hypothesis (CBH), originally developed based on the relatively high prosocial tendencies of cooperatively breeding common marmosets [3]. The hypothesis states that owing to the social requirements of cooperative breeding (in particular, helping), enhanced prosocial attitudes have evolved convergently in both humans and marmosets.

The most convincing evidence for the CBH so far is a comparative study of 15 primate species that showed that species-specific prosocial tendencies in a group service paradigm were best explained by the degree of allomaternal care [4]. However, a major criticism of the CBH in general, and this study specifically is that it focuses only on primates [5], whereas—if true—the same pattern should be apparent in other lineages. Moreover, the only cooperative breeders in that sample, apart from humans, were all members of the family Callitrichidae, and independent evolution of prosociality in these New World monkeys and humans due to alternative causes cannot be excluded.

Birds are a very interesting clade to test the CBH, as about 9% of all extant bird species are cooperative breeders [6] and consequently, they allow testing the hypothesis in a lineage other than primates. From a comparative perspective, the corvid is of particular interest, as corvids have similar neuron counts to many primates [7] and show similarly complex cognitive traits [8]. So far, studies investigating prosociality have focused on territorially breeding ravens (*Corvus corax*), and colonially breeding jackdaws (*C. monedula*); while ravens were indifferent to benefitting others (e.g. [9]), jackdaws provided food to their partners in a two-choice task, but only when recipients first showed interest in the side where food was available [10].

Here we examined prosociality in azure-winged magpies, East-Asian corvids that are colonial cooperative breeders. Helping is performed by both related and unrelated group members in naturalistic contexts and is relatively flexible with regard to who helps when [11]. To study their prosocial tendencies, we used a group service paradigm comparable to that used with primates [12].

## 2. Material and methods

### (a) Subjects

We tested two groups of azure-winged magpies, a non-breeder group (NB; *N* = 5) housed at Haidlhof Research Station, and a breeder group (B; *N* = 4) housed at the Animal Care Facility of the Department of Cognitive Biology (table 2; for keeping conditions, see electronic supplementary material, SM1). All subjects except one, which was born at our facility, originated from zoo populations and their relatedness was unknown. The animals were tested in their social group prior to their first feeding of the day. High-quality food reward (i.e. mealworms, crickets) was used in the experiment. Water was available ad libitum.

### (b) Apparatus

**Figure 1.**
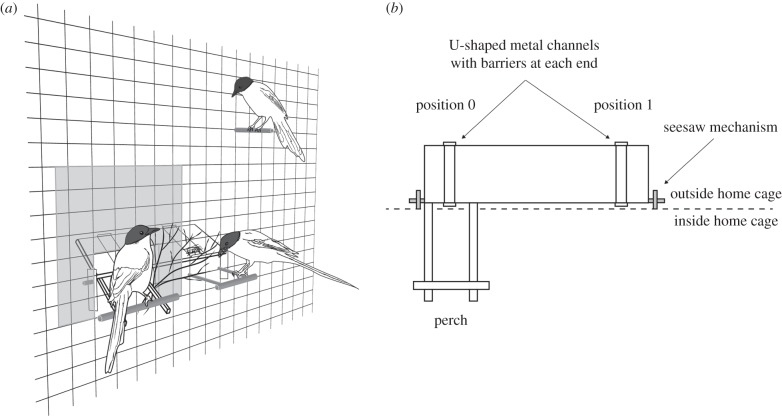
(*a*) Experimental set-up as seen from the inside of the aviary; see electronic supplementary material, SM2 for a video illustration. (*b*) Schematic of the apparatus with location of positions 0 and 1.

We used an apparatus with a seesaw mechanism. It consisted of a board outside the aviary and two sticks reaching through the wire mesh into the aviary on one side of the board with a perch fixed at their end. The apparatus's mechanism was balanced so that in the starting position the perch on the inside pointed up and the board on the outside pointed down. When a bird landed on the perch, its weight moved the seesaw down. As soon as the bird left the perch, the apparatus automatically moved back to its original position. Near the other side of the board, inside the aviary, were branches that were not connected to the apparatus's seesaw mechanism (figure 1*a*).

There were two positions for putting food on the board: one in front of the perch (position 0) and one on the other side of the board (position 1) out of reach from the perch. If food was placed in position 0, a subject could deliver food to itself by landing on the perch, after which the food slid towards the wire mesh and into reach. If food was placed in position 1 and a bird landed on the perch, it could not obtain the food itself. However, if it stayed on the perch, it made food available to the group (figure 1*b*; electronic supplementary material, SM2).

### (c) Procedure

Testing took place Apr–Nov 2015 and Nov 2015–May 2016 in the groups NB and B, respectively. We replicated the procedures of Burkart & Van Schaik [12] as closely as possible, while making two modifications: first, owing to greater object-related neophobia in corvids [13], we added an additional habituation phase (phase 0; see the electronic supplementary material, SM1); second, we reduced the number of trials to 25 trials per session in each phase to avoid satiating the birds. Consequently, the experiment consisted of six consecutive phases in a fixed sequence: three habituation/training phases and three test phases.

In each habituation and training phase (i.e. phases 0, I, and III), all animals had to meet a specific criterion in order for the whole group to proceed to the next phase (table 1). One bird in group B (i.e. Amidala) did not reach criterion in phase III after more than 40 training sessions, but we decided to nevertheless move on to the test phase.

**Table 1.** Procedures of the six phases of the experiment.

phase	name	seesaw mechanism	food position	sessions
phase 0	habituation to the apparatus^a^	fixed	in front of the apparatus	until criterion
phase I	habituation to the procedure	fixed	position 0 and position 1 (alternating sessions)	until criterion
phase II	food distribution assessment^b^	fixed	position 1	two test sessions
phase III	training	released	position 0	until criterion
phase IV	group service	released	position 1	five test sessions and five empty sessions
phase V	blocked control	released	position 1	five blocked sessions and five empty blocked sessions

^a^Not part of the original procedure of Burkart & Van Schaik [12].

^b^Originally called ‘social tolerance’.

In the group service test (phase IV), food was placed in position 1, so that a bird landing on the perch could only make food available to the group, not to itself. On alternating days, we conducted empty control sessions, which were identical to test sessions except that no food was placed on the apparatus. In the blocked control (phase V), access to food in position 1 was blocked with a fine net to test whether landing was simply elicited by the presence of food. We added motivation trials where food was placed in position 0 in all sessions of phases IV and V to ensure that the birds were still motivated to participate in the experiment (table 1). For the analysis, we used only the data from the last two sessions (sessions 4 and 5) of each condition, because by then each bird had had the opportunity to learn about the consequences of operating the apparatus. Additionally, we retested both groups with two test sessions and two empty control sessions in Apr–May 2016 (see also [14]). None of the groups had received any training with the apparatus in-between original test and retest. All sessions were video-recorded. For detailed procedures, see electronic supplementary material, SM1.

## 3. Results

To calculate the evenness of food distribution as a proxy for social tolerance in the group we used Pielou's *J*′, which is expressed as a number between 0 and 1 (ranging from 0, indicating maximal inequality to 1, indicating a completely equal distribution [12]). The evenness of distribution assessed in phase II was high in both groups (NB: Pielou's *J*′ = 0.74; B: *J*′ = 0.85; table 2).

**Table 2.** Subjects' individual results in phases II (food obtained by itself), IV and V (food received from and provided to others, landings on the perch). Data for the subject that did not reach criterion in phase III are given in italics. YOB, year of birth.

	individual			phase II	food phase IV		landings phase IV & V		
name	group	sex	YOB	obtained	received	provided	test	empty	blocked
Boots	NB	M	2012	2	38	0	0	1	0
Yoda	NB	M	2013	19	10	1	8	0**	0**
ObiWan	NB	M	2014	18	0	21	26	6***	8***
Mon	NB	F	2014	10	1	27	38	3***	3***
Padme	NB	F	2014	0	0	0	2	3	3
Han	B	M	2014	3	8	32	34	13***	12***
Leia	B	F	2014	22	24	0	0	3	3
Chewie	B	F	2015	18	0	16	17	7*	16
*Amidala*	*B*	*F*	*2012*	*7*	*16*	*0*	*0*	*0*	*0*

Fisher's exact tests: *p < 0.05, **p < 0.01, ***p < 0.001.

**Figure 2.**
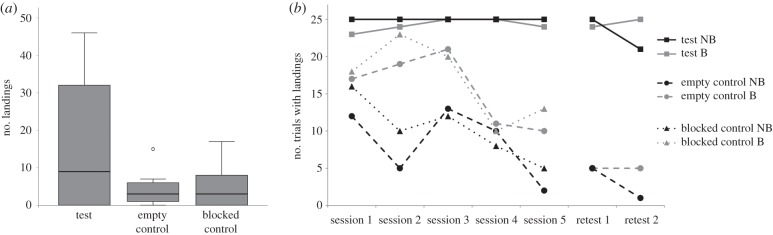
(*a*) Median, inter-quartile range and range of landings in test, empty and blocked control; (*b*) number of trials with landings across all five sessions of test, empty and blocked control, and of the retest of test and empty control.

Throughout the experiment, magpies delivered food to others at very high rates. In the last two test sessions, successful deliveries occurred in 98% and 96% of the trials in the groups NB and B, respectively. In only four of these 97 trials, a bird was already sitting in position 1 when another bird delivered the food. Figure 2*a* shows that there was a trend that the birds landed more often in test sessions than in empty control (Wilcoxon, Holm–Bonferroni corrected: *T*^+^ = 25, *n* = 9, *p*_1-sided_ = 0.076) and blocked control sessions (*T*^+^ = 14, *n* = 9, *p*_1-sided_ = 0.052). Individually, five birds landed significantly more often in test sessions than in empty control sessions and four birds landed significantly more often in test sessions than in blocked control sessions (table 2). The other birds (two per group) hardly landed on the provisioning perch at all (table 2).

Notably, both groups showed a sustained rate of landings across all five test sessions, whereas the rate of landings decreased in both controls. When we retested both groups, birds landed at similar rates (NB: 92%, B: 98%) compared with the original test and they landed significantly more often in the test trials than in the empty control trials (NB: 12%, B: 20%; *T*^+^ = 25, *n* = 9, *p*_1-sided_ = 0.037; figure 2*b*).

## 4. Discussion

Here we show that food was generally distributed evenly in the two groups of azure-winged magpies, which is considered to indicate high levels of social tolerance between group members [4], and evenness measures were in the range of cooperatively breeding primates [12]. Moreover, in the group service test five out of nine birds landed more on the provisioning perch in the test than in the controls, whereas the other birds hardly landed at all. In the last two sessions the providing birds were very fast, thereby not giving the remaining birds enough time to land on the provisioning perch. Consequently, owing to the ceiling performance of the providers, not all individuals that may have been motivated to provide food also had the possibility to do so. Additionally, the relatively small sample size may explain the lack of significance on the group level. Nevertheless, provisioning rates of these five birds in the test were very high (almost 100%; cf. cooperatively breeding primates [4]) and, in contrast with the controls, remained so over all sessions (figure 2*b*). Moreover, this provisioning was not due to local or stimulus enhancement (cf. [10]), as provisioning birds generally landed first and then waited for another bird to retrieve the reward. Consequently, we conclude that the azure-winged magpies showed proactive prosociality.

A criticism of the original procedure [12] was that the blocked control sessions were conducted after the test sessions, and subsequently order effects (e.g. lack of motivation) may come into play [5]. Therefore, we retested our birds and found comparable numbers of landings during this retest, rendering order effects unlikely (cf. primates [4,14]).

That prosocial tendencies were found in a cooperatively breeding corvid suggests that the CBH may apply not only to primates [4] but also to other taxa, specifically, as other corvids that are not cooperatively breeding species so far have not shown evidence for proactive prosociality [9,10]. However, to truly test the CBH these and additional corvid species need to be tested under comparable conditions, and we conclude that the adapted group service paradigm described here is a promising way to do so. Also, in our relatively small groups, cooperative breeding comparable to that in the wild [11] does not occur. It would be interesting to investigate whether the status of being a breeder or helper influences prosocial tendencies in more naturalistic settings.

## Supplementary Material

SM1 - Supplementary Methods

## Data accessibility

Raw data are provided in table 2.
