# Supplementary material for: Proactive prosociality in a cooperatively breeding corvid, the azure-winged magpie (Cyanopica cyana)
Source: Biol Lett. 2016 Oct;12(10):20160649. doi: 10.1098/rsbl.2016.0649 (PMC5095199; doi:10.1098/rsbl.2016.0649)
Supplement: SM1 - Supplementary Methods [file rsbl20160649supp1.pdf]

“Proactive prosociality in a cooperatively breeding corvid, the azure-winged magpie (*Cyanopica cyana*)”

Lisa Horn, Clara Scheer, Thomas Bugnyar & Jorg J.M. Massen

Supplementary Material

(a) Animal keeping conditions

Eight azure-winged magpies were obtained from zoo populations (7 from Blijdorp Zoo, Rotterdam, Netherlands; 1 from Ostrava Zoo, Ostrava, Czech Republic) in 2014. One bird was born at our facility in 2015. The birds were maintained in two groups in two different locations. One group (3m/2f) was kept at Haidlhof Research Station, Bad Vöslau in an outdoor aviary (5m x 3m x 3m) with partial roof covering. The other group (1m/3f) was kept at the Animal Care Facility of the UZA I, Vienna in an outdoor aviary (6m x 3m x 3m) that was completely covered with a semi-transparent roof. Both aviaries used fine-grained sand as substrate and were equipped with fixed and swinging branches, live plants, stones, woodchips and gravel for caching food, a birdbath, and other enrichment objects. The animals were fed daily with different fruits, insects, and seeds. Water and pellets (“Beo komplet”, NutriBird) were provided ad libitum. Vitamin supplements and meat or egg were provided every second week. Daylight was the main source of lighting.

(b) Supplementary procedure

The experiment consisted of six consecutive phases in a fixed sequence: three habituation/training phases and three test phases.

Phase 0 – Habituation to the apparatus

The apparatus was installed in the home aviary. After two weeks the seesaw mechanism was fixed with the provisioning perch pointing downwards. A food bowl was mounted in front of the perch on the inside of the aviary (Position 0; main document, Figure 1b). In each session, the bowl was filled with mealworms and the birds were video-recorded for thirty minutes. A bird reached criterion when it had landed on the perch and fed from the bowl at least five times.

Phase I – Habituation to the procedure

The seesaw mechanism was still fixed with the perch in a downward position, so that food (i.e. crickets) placed on the board would automatically slide to the wire mesh and into the birds' reach. On alternating days crickets were provided either in position 0 or 1. In each trial the experimenter called the birds' attention and placed one cricket on the board. The next trial started after a bird obtained the food or after a maximum of two minutes. If a bird took the cricket, the experimenter placed the next cricket on the board. If no bird took the cricket, the experimenter called the birds' attention again, lifted the same cricket and placed it back on the board. A session ended after 25 trials or when none of the birds landed on the perch for three consecutive trials. If a bird (or several birds) started monopolizing the apparatus, this bird (these birds) was (were) distracted or temporarily separated from the group. A bird reached criterion when it had taken at least ten pieces of food in a minimum of five sessions.

Phase II – Food distribution assessment (Test phase)

The seesaw mechanism was still fixed with the perch in a downward position. The experimenter put 25 crickets on the apparatus in position 1, one at a time and called the birds' attention each time. After the food was taken the experimenter placed the next cricket on the board. Two sessions of the food distribution assessment were conducted on two consecutive days. We recorded how many crickets each bird obtained.

Phase III – Training

In this phase the birds learnt to move food towards the wire mesh by landing on the perch. Food was always placed in position 0. To facilitate learning, the seesaw mechanism was first partially released – so that the perch moved only slightly – and food was placed close to the wire mesh. When each bird had obtained food from the apparatus at least once, the mechanism was released further. In the final step the seesaw mechanism was completely released and the food was placed at the other end of the board.

In each trial the experimenter called the birds' attention and placed one cricket on the board. The next trial started after a bird obtained the food or after a maximum of two minutes. A session ended after 25 trials or when none of the birds landed on the perch for three consecutive trials. Again, if a bird started monopolizing the apparatus, this bird was distracted or temporarily separated from the group. A bird reached criterion when it had taken at least

ten pieces of food in a minimum of five sessions with the seesaw mechanism completely released.

Phase IV – Group service (Test phase)

In this phase, the apparatus' seesaw mechanism was completely released. We conducted five test sessions and five empty control sessions on alternating days.

In a regular trial of a test session, a cricket was placed in position 1. Additionally, each session comprised motivation trials with food in position 0 in the very beginning of the session and after every fifth regular trial. Each session consisted of 25 regular and 6 motivation trials. In each trial the experimenter called the birds' attention and placed one cricket on the board. The next trial started after a bird obtained the food or after a maximum of two minutes.

The empty control sessions were identical to the test sessions, except that in the regular control trials no food was placed on the board. In these trials, the experimenter approached the apparatus and pretended to leave a cricket in position 1, while calling the birds' attention. Control sessions also comprised motivation trials with food in position 0. Each session consisted of 25 regular and 6 motivation trials.

For each trial, we recorded which animal(s) landed on the perch in position 0 (i.e. moved the seesaw mechanism) and which animal(s) landed in front of position 1. Additionally, we recorded which animal obtained the cricket, which animal provided the cricket and whether a bird was present in position 1 prior to another bird providing the cricket.

Phase V – Blocked control (Test phase)

In this phase the access to position 1 was blocked with a fine-meshed net, so that no food could be obtained in this position. Otherwise, the procedure was exactly the same as in group service and we conducted five test sessions and five empty control sessions on alternating days. For each trial, we recorded which animal(s) landed on the perch in position 0 and which animal(s) landed in front of position 1.
